# Acute coronary syndrome caused by extrinsic coronary compression from an aortic root abscess in a patient with mechanical aortic valve endocarditis: a case report and literature review

**DOI:** 10.1093/ehjcr/ytaa483

**Published:** 2020-12-28

**Authors:** George Joy, Michael Lewis, Stephen Furniss

**Affiliations:** 1 Cardiology Department, St Bartholomew's Hospital, Barts Heart Centre, West Smithfield, London EC1A 7BE, UK; 2 Department of Cardiac Surgery, Brighton and Sussex Medical School, 94 N - S Rd, Falmer, Brighton BN1 9PX, UK; 3 Cardiology Department, East Sussex Healthcare NHS Trust, Kings Drive, Eastbourne, East Sussex BN21 2UD, UK

**Keywords:** Aortic root abscess, Coronary compression, Infective endocarditis, Case report

## Abstract

**Background:**

Extrinsic coronary compression is an extremely rare complication of aortic root abscess formation and can manifest as an acute coronary syndrome in infective endocarditis. Optimal management strategies are unknown and therefore illustrative case reports may be informative.

**Case summary:**

We describe a 63-year-old man with a background history of a mechanical aortic valve who developed sepsis due to *Escherichia coli* bacteraemia from a presumed urinary source. He suddenly deteriorated with cardiogenic shock and anterior ST-segment elevation myocardial infarction on Day 16 and received emergency percutaneous coronary intervention for severe stenoses of left anterior descending and diagonal arteries. A transoesophageal echocardiogram 2 days later demonstrated a large aortic root abscess. He was transferred for emergency surgery which revealed a large aortic abscess surrounding the left main stem confirming extrinsic coronary compression. He received a redo tissue aortic valve replacement and repair of his abscess cavity.

**Discussion:**

We describe a case where percutaneous coronary intervention and emergency surgery was used to treat extrinsic compression from an aortic root abscess; a complication that is associated with a high mortality. This is also a rare case of *E. coli* causing prosthetic valve endocarditis. We also explore the findings of 11 previous cases of extrinsic coronary compression from aortic root abscess.

Learning points

- Early transoesophageal echocardiogram is crucial in a patient with sepsis of unknown origin and a prosthetic intracardiac valve.
- *Escherichia coli* bacteraemia does not necessarily exclude endocarditis as a source of sepsis.
- This case should alert clinicians on the risk of coronary complications in aortic valve endocarditis.
- This case also describes the first successful use of percutaneous coronary intervention in this rare clinical phenomenon with overall positive outcomes for the patient.

## Introduction

Acute coronary syndrome (ACS) occurs in 2.2% of infective endocarditis (IE) with a mortality of 27% based on registry data.^1^ Acute coronary syndrome is associated with a higher mortality and risk of heart failure in the setting of IE. Extrinsic compression from aortic root abscess is extremely rare being present in 11 previous case reports and only 1 of the 26 patients studied in a retrospective study of 1210 patients giving an incidence of 4% of patients with ACS in IE.^1^ The commonest mechanism of ACS in IE is septic emboli. Other causes include mycotic aneurysm formation, severe aortic regurgitation, external compression, or aortic valve vegetations obstructing coronary ostia.^1^*Escherichia coli* has infrequently been associated with prosthetic valve endocarditis. We describe a 63-year-old man who suffered with an aortic root abscess of his mechanical aortic valve causing extrinsic coronary compression.

## Timeline

**Table:** 

Day 1	Hospital admission with fevers, dry cough, and atypical chest pain.
Day 2	Blood and urine cultures grew extended spectrum beta-lactamase *Escherichia coli*. Treated for sepsis of presumed urinary source with intravenous antibiotics.
Days 3–15	Ultrasound abdomen and whole-body computed tomography was negative for infective source.
Day 16	Sudden deterioration with ST-segment elevation myocardial infarction and cardiogenic shock. Coronary angiogram revealed severe stenoses of his left anterior descending and diagonal for with he received primary percutaneous coronary intervention.
Day 18	Ventricular tachycardia arrest cardiac arrest in intensive treatment unit for which he was successfully cardioverted.
Day 19	Transoesophageal echocardiogram confirmed an aortic root abscess and mechanical aortic valve endocarditis. Emergency surgery with a redo tissue aortic valve replacement and abscess cavity repair.

## Case presentation

**Figure 1:**
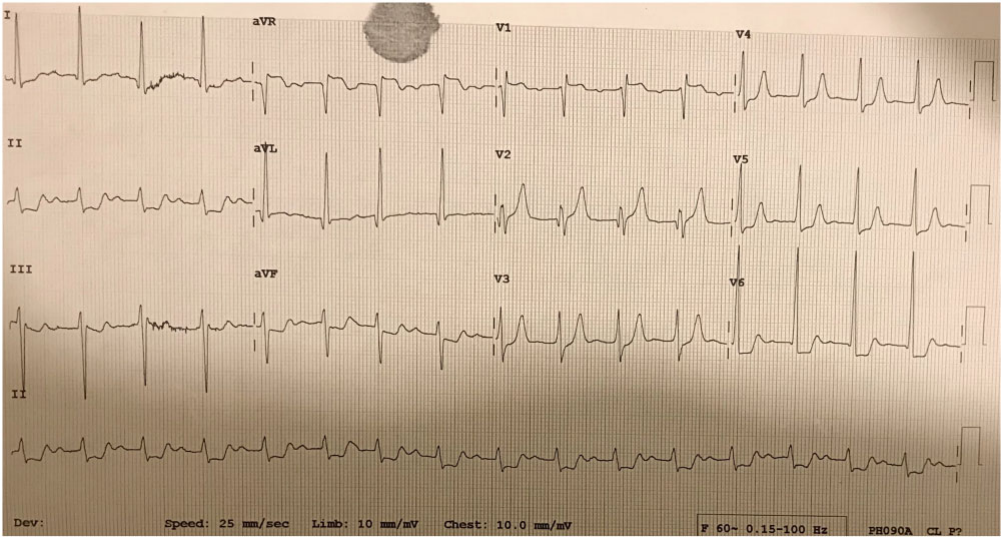
Electrocardiogram showing sinus tachycardia and ST elevation in aVR, V1, and V2.

**Figure 2:**
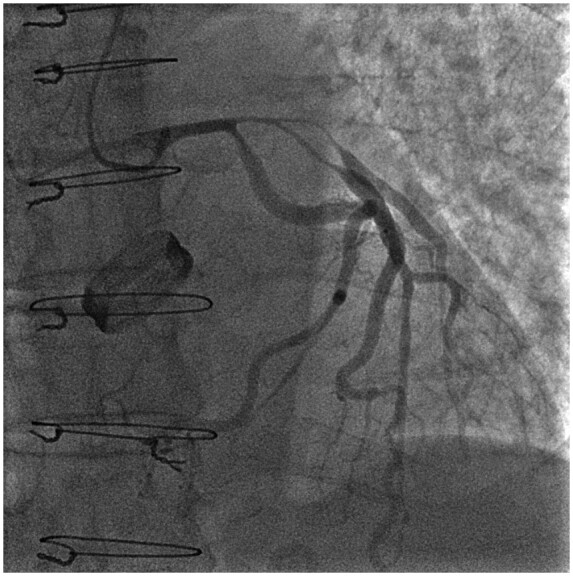
Diagnostic angiography (post-intracoronary nitrate) shows Medina 1:1:1 long tubular severe stenosis of left anterior descending and D1 (red arrow) consistent with external compression. The lesion also involves left main stem and ostium of left circumflex.

**Figure 3:**
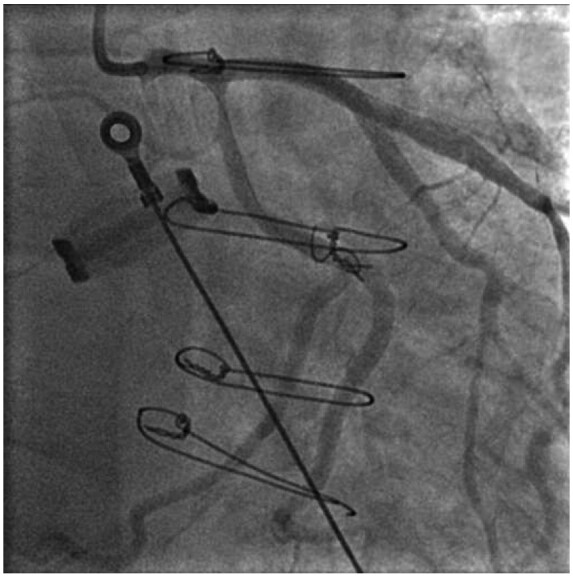
2× drug-eluting stent to left anterior descending (resolute onyx 3.5 × 34 mm, resolute onyx 2.5 × 8 mm), 1× drug-eluting stent to Proximal diagonal lesion (resolute onyx 2.0 × 30 mm). Final Result as shown—TIMI 3 flow down left anterior descending and diagonal. Proximal left circumflex lesion left for a possible staged procedure.

**Figure 4:**
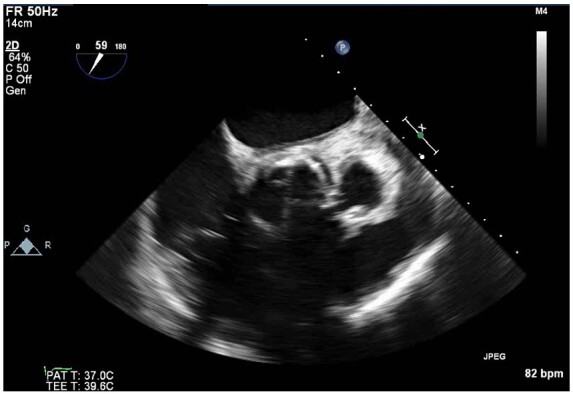
Transoesophageal echocardiogram mid-oesophageal short-axis aortic valve view showing peri-annular aortic root abscess.

**Figure 5:**
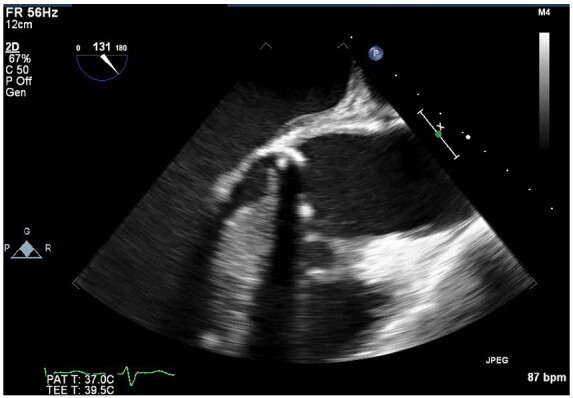
Transoesophageal echocardiogram mid-oesophageal long-axis view showing peri-annular aortic root abscess.

**Figure 6:**
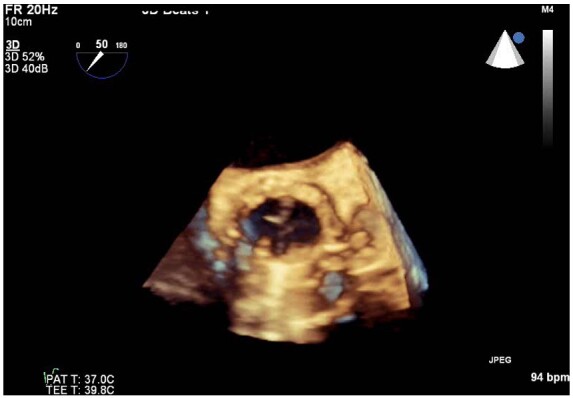
Three-dimensional transoesophageal echocardiogram of short-axis view with showing peri-annular aortic root abscess.

A 63-year-old man was admitted to the emergency department with fevers, dry cough, and transient non-exertional central chest ache. He had a history of mechanical aortic valve replacement (AVR) 15 years earlier due to rheumatic heart disease for which he took warfarin. On examination, he appeared diaphoretic and was febrile (>40°C) but was haemodynamically stable with no respiratory compromise. His chest sounds were clear and his heart sounds revealed his mechanical second heart sound with no added sounds. There were no peripheral stigmata of IE. His blood tests showed deranged liver function tests, C-reactive protein 197 mg/L (normal 0–10 mg/L) and international normalized ratio (INR) >10 (target range 2–3). His dipstick urine test was strongly positive for blood. His chest radiograph revealed his aortic valve prosthesis but no consolidation or pulmonary oedema. His blood and urine cultures grew extended spectrum beta-lactamase *E. coli* and he was commenced on intravenous Meropenem for a presumed urinary source of sepsis. He continued to spike daily fevers and an ultrasound abdomen and a computed tomography (CT) abdomen and pelvis did not reveal an alternative source of infection. He became acutely unwell with respiratory failure and tachycardia on Day 16 and his electrocardiogram showed sinus tachycardia and anterior ST-elevation (*Figure 1*). He was taken immediately to the cardiac catheter lab and was found to have diffuse tapering stenosis of his left anterior descending (LAD), diagonal, and circumflex vessels. He had percutaneous coronary intervention (PCI) to LAD and diagonal with three drug-eluting stents (*Figure2, Supplementary Material*). He became extremely hypotensive in cardiogenic shock and was commenced on a dobutamine infusion and taken to the high dependency unit. On Day 18, he had a pulseless ventricular tachycardia arrest requiring two cycles of cardiopulmonary resuscitation and he was intubated and managed on the intensive treatment unit. He received a transoesophageal echo which showed an aortic root abscess and aortic valve endocarditis (*Figures 3–6, Supplementary Material*). He was extubated successfully and transferred to a cardiothoracic centre for emergency surgery after rapid pharmacological reversal of warfarin. The aorta was opened and the mechanical valve and infected tissue were explanted. A large abscess cavity surrounding the left main stem (LMS) was revealed confirming extrinsic coronary compression as a cause of his ST-segment elevation myocardial infarction (STEMI). The right ventricular outflow tract was opened to decompress the abscess cavity. The abscess had formed a left coronary to right ventricular (RV) fistula which was oversewn. The large defect in the aorta was repaired with a bovine pericardial patch. A 23-mm Magna (Edwards Lifesciences) tissue aortic valve was placed. The culture of the explanted valve revealed *E. coli*. His transthoracic echocardiogram at discharge showed an ejection fraction of 25% with a well seated AVR. Due to spontaneous contrast in his left ventricle (LV), he was felt to have a high risk of future LV thrombus and was commenced on rivaroxaban and aspirin. He was successfully discharged following a prolonged inpatient stay and progressed well with rehabilitation. At the time of writing, he is active and lives independently. Unfortunately, he continues to suffer with shortness of breath [New York Heart Failure Association (NYHA) 3] secondary to ischaemic cardiomyopathy. A cardiac magnetic resonance study performed 2 years following his admission shows a large LAD territory and right ventricular chronic myocardial infarction causing severely impaired biventricular systolic function. He receives optimal medical management for heart failure and is scheduled for cardiac resynchronisation device therapy.

## Discussion

We describe a case where PCI and emergency surgery was used to treat extrinsic compression from an aortic root abscess; a complication that is associated with a high mortality. This is the first case described of this complication in a mechanical aortic valve. The low initial suspicion for IE due to positive urinary cultures and an atypical organism incurred delays to definitive management and is a crucial learning point of this case. Although *E. coli* endocarditis is extremely rare, it is an important differential diagnosis in the setting of *E. coli* bacteraemia and patients with a prosthetic valve who are inherently high risk. This case is also unique as the patient survived several catastrophic complications of endocarditis; aortic root abscess, STEMI, and cardiogenic shock.

The pattern of diffuse tapering stenosis involving the LMS, proximal LAD/diagonal bifurcation and circumflex matches the angiographic appearance of other reported cases of extrinsic compression from abscess that was confirmed with CT.^2–5^ A CT scan was not performed due to his moribund clinical status and urgency of his surgery. Transoesophageal echocardiogram (TOE), however, demonstrated the abscess was in close proximity to the left coronary artery origin and compression was later confirmed at surgery. At the time of surgery, the abscess had formed an RV fistula which was not present on his TOE following his STEMI, suggesting it was a new progression of his abscess rather than a cause of his STEMI. His PCI was a high-risk procedure due to risks of propagating potential septic emboli and the prospect of placing coronary stents during active endocarditis. However, PCI was deemed necessary in the setting of cardiogenic shock and STEMI. One previous case does describe the attempted use of bare-metal stents in a similar circumstance; however, the patient died from cardiogenic shock, highlighting the life-threatening nature of this complication.^2^

We identified 11 contemporary cases of extrinsic coronary compression from aortic root abscess on literature search (*Table 1*).^2–12^ Demographics were widespread in terms of age (ranging from 22 to 82 years). Nine males were affected. Four were caused by prosthetic aortic valve endocarditis; however, six of eight native valve endocarditis had known aortic valve disease prior to admission. No patient had a mechanical aortic valve. Time of onset from symptoms of endocarditis to clinical syndrome caused by the coronary compression ranged from a few days to 2 months. The proximal left coronary artery was involved in all but one case where the aortic root abscess was compressing the right coronary artery. It is clear from the literature that the clinical syndrome is associated with critical illness (five had cardiogenic shock and one resulted in sudden cardiac arrest) and mortality (seven patients died by the time the case was described). *Staphylococcus aureus* was the causative organism in seven patients; the remaining were *Staphylococcus epidermis*, *Enterococcus faecalis*, and two patients had negative blood cultures.

**Table 1:** Summarized clinical features, angiogram findings, and outcomes of all patients identified in literature review with extrinsic coronary compression from aortic root abscess

Author	Age/ gender	Past medical history	Clinical presentation, timing of ACS from symptoms of IE and organism identified	Angiographic and other imaging findings	Cardiogenic shock and management	Surgical management/PCI	Outcome	Echo and other imaging findings and aortic root abscess description
Campanile *et al.*^2^	82 F	AF, Chronic renal failure, anaemia, bioprosthetic AVR 4 years previously	Anterior STEMI—1 month *S. aureus*	Severe narrowing and complete occlusion of proximal LAD, long tubular narrowing of circumflex	Yes—inotropes, vasopressors, NIV	Two bare-metal stents to LAD and proximal CX	Pt died within 24 h despite intensive treatment		
Dean *et al.*^3^	44 M	Bicuspid aortic valve and mild AS	NSTEMI—2 weeks *Staphylococcus aureus*	LAD—long smooth stricture, obstructed intermediate.	Yes—intra-aortic balloon pump, inotropes	Abscess cavity (3 cm) drained and vein graft placed to LAD	Pt Died	CT—non-enhancing mass anterior and to the left immediately above aortic valve
Misuraca *et al.*^6^	79 M	Severe AS, coronary artery disease, COPD, smoking	NSTEMI—unspecified time on onset *Enterococcus faecalis*	LMS size reduction, ostial stenosis of LAD and circumflex	No	Cardiac arrest during angiogram, intubated and ventilated, bioprosthetic valve and vein grafts to LAD and obtuse marginal	Pt well at 1 year follow-up	Aortography—paravalvular opacification of a globular cavity
Cowan *et al.*^7^	61 M	7 months previously, aortic root abscess with tricuspid and mitral valve vegetations, treated with bioprosthetic valve	AF and anterior ST elevations—4 weeks *S. aureus*	Eccentric 90% stenosis of LMS	No	Emergency surgery, debridement of abscess, aortic root homograft	Completed 6 weeks of iv antibiotics, follow-up angiogram showed unobstructed coronaries	Aortic root abscess extending from the non to the left coronary sinus
Tomoaia *et al.*^4^	48 M	Recent dental treatment, gastric ulcer	NSTEMI—2 weeks *Methicillin-resistant S. aureus (MRSA)*	Proximal circumflex and LAD narrowing	Yes—inotropes	Planned for emergency surgery, however, cardiac arrest prior to procedure	Pt died	Perforated left coronary cusp, aortic root abscess containing two cavities located on the posterior and lateral sides of aortic root
Namboodiri *et al.*^8^	22 M	Medically treated for *S. aureus* AoV endocarditis 4 months prior	stable angina (shortness of breath and exertional chest pain)—2 months *sterile blood cultures*	Distal LMS, proximal LAD and LCX smooth long subtotal occlusion with retrograde filling	No	Pericardial patch exclusion of abscess cavity, LIMA to LAD, SVG to OM, AoV not replaced	pt well at 18-month follow-up, mild AR only	Multiloculated aortic root abscess near the left coronary sinus and possible compression of L MS
Jenny and Almanaseer^5^	73 F	Aortic stenosis, atrial fibrillation, diabetes, gastrocutaenous fistula, sacral decubitus ulcer	Anterior STEMI—unspecified *S. aureus*	Proximal RCA tubular stenosis, possible flap, CT—contrast collection along anterior R aortic annulus	No	Declined surgical intervention	Died	Aortic root abscess extending anteriorly into the R sinus of valsalva
El Kandoussi *et al.*^9^	72 M	Rheumatic heart disease, hypertension	NSTEMI—2 month *S. aureus*	Left main/ LAD narrowing. CT—mycotic aneurysm of superior mesenteric artery	No	Emergency surgery—obliteration of abscess, bioprosthetic aortic valve replacement, LIMA to LAD	pt died of multiorgan failure	Large circumferential peri-aortic root abscess
Allan *et al.*^10^	52 M	Bioprosthetic AoV replacement	NTEMI—9 days *S. aureus*	No angiogram	Yes—inotropes, mechanical ventilation	Died 12 h following admission, before planned emergency surgery	pt died	Periaortic abscess causing compression of LMS, multiple vegetations of aortic bioprosthesis. RCA aneurysmally dilated. Severe LVSD
Clarke and Forfar^11^	68 M	Mixed aortic valve disease	Unstable angina—4 weeks *culture negative*	Aortography—severe AR, proximal LAD, and CX artery with a tapering contour	No	Emergency surgery—large anterior aortic root abscess destroying left and right coronary cusps. Bioprosthetic AoV replacement, SVG to LAD	Represented 15 months later with dehiscence of the Dacron graft and right coronary button. Died of sternal wound infection	Aortography—severe aortic regurgitation and distorted aortic root
Atik *et al.*^12^	46 M	Bicuspid aortic valve with severe AS undergoing bioprosthetic AVR	NSTEMI—a few days *Staphylococcus epidermis*	LMS 80% long tapering lesion	Yes—IABP, inotropes, mechanical ventilation	Urgent AoV replacement, large anterior aortic root abscess debrided, aortic root replaced with homograft, closure of aortic to RA Fistula, SVG to LAD performed.	Alive and well 36 months after operation, normal LV function, NYHA 1	Vegetations attached to the prosthesis aortic root abscess, aortic to RA fistula, LVEF 36%

ACS, acute coronary syndrome; AF, atrial fibrillation; AoV, aortic valve; AR, aortic regurgitation; AS, aortic stenosis; AVR, aotric valve replacement; COPD, chronic obstructive pulmonary disease; CT, computed tomography; CX, circumflex; IABP, intra-aortic balloon pump; IE, infective endocarditis; IV, intravenous; LAD, left anterior descending; LIMA, left internal mammary artery; LMS, left main stem; LV, left ventricle; LVEF, left ventricular ejection fraction; NIV, non-invasive ventilation; NSTEMI, non-ST-segment elevation myocardial infarction; PCI, percutaneous coronary intervention; Pt, patient; RA, right atrium; STEMI, ST-segment elevation myocardial infarction; SVG, saphenous vein graft.

The optimal strategy of reperfusion in ACS during IE remains unclear due to the rarity of the condition and paucity of outcome data. Percutaneous coronary intervention may be complicated by LMS or distal embolization of vegetation. Percutaneous coronary intervention can also lead to the development of mycotic aneurysm and incurs risks of dissection, perforation, or post-stent stenosis. It may, however, be considered in critical illness as with our patient and should be contemplated on a case-by-case basis. Aspiration thrombectomy has been successfully used in previous cases with the added benefit of culture and sensitivity of aspirated vegetations.^13^ Thrombolysis is associated with negative outcomes in the presence of IE due to the high risk of intracranial haemorrhage from subclinical cerebral emboli.^1^

Prosthetic valve *E. coli* endocarditis is a non-HACEK (haemophilus, aggregatibacter, *Cardiobacterium hominis*, *Eikenella corrodens*, and kingenella) gram-negative bacilli infection that is extremely rare being described mainly in case reports. Incidences have been quoted as low as 0.51% of all endocarditis and are associated with older age, women and states of immunocompromise such as diabetes, haemodialysis, and malignancy.^14^ It does occur more commonly in prosthetic valves and previous systematic review reveals 52% has a urinary source as with our patient and up to a third are considered nosocomial in nature.^14^^,^^15^ The mortality is high (21%).^15^ Registry studies suggest the occurrence of intracardiac abscess with non-HACEK infection to be 42%.^14^

## Conclusion

This case highlights the rare but catastrophic complication of extrinsic coronary compression that can occur with aortic root abscess and how percutaneous coronary intervention and emergency surgery was used in its management.

## Lead author biography

**Figure:**
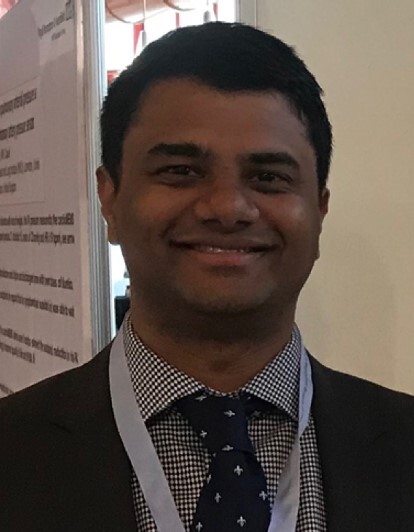


Dr George Joy is a research fellow in cardiac imaging at Barts Heart Centre. His research interests include interventional cardiology and advanced imaging in cardiomyopathy.

## Supplementary material

Supplementary material is available at *European Heart Journal - Case Reports* online.

**Slide sets:** A fully edited slide set detailing this case and suitable for local presentation is available online as Supplementary data.

**Consent:** The authors confirm that written consent for submission and publication of this case report including images and associated text has been obtained from the patient in line with COPE guidance.

**Conflict of interest:** none declared.

**Funding:** none declared.

## Supplementary Material

ytaa483_Supplementary_Data
