# Supplementary material for: Acute coronary syndrome caused by extrinsic coronary compression from an aortic root abscess in a patient with mechanical aortic valve endocarditis: a case report and literature review
Source: Eur Heart J Case Rep. 2020 Dec 28;5(1):ytaa483. doi: 10.1093/ehjcr/ytaa483 (PMC7850617; doi:10.1093/ehjcr/ytaa483)
Supplement: ytaa483_Supplementary_Data [file ytaa483_supplementary_data.pptx]

## Slide 1
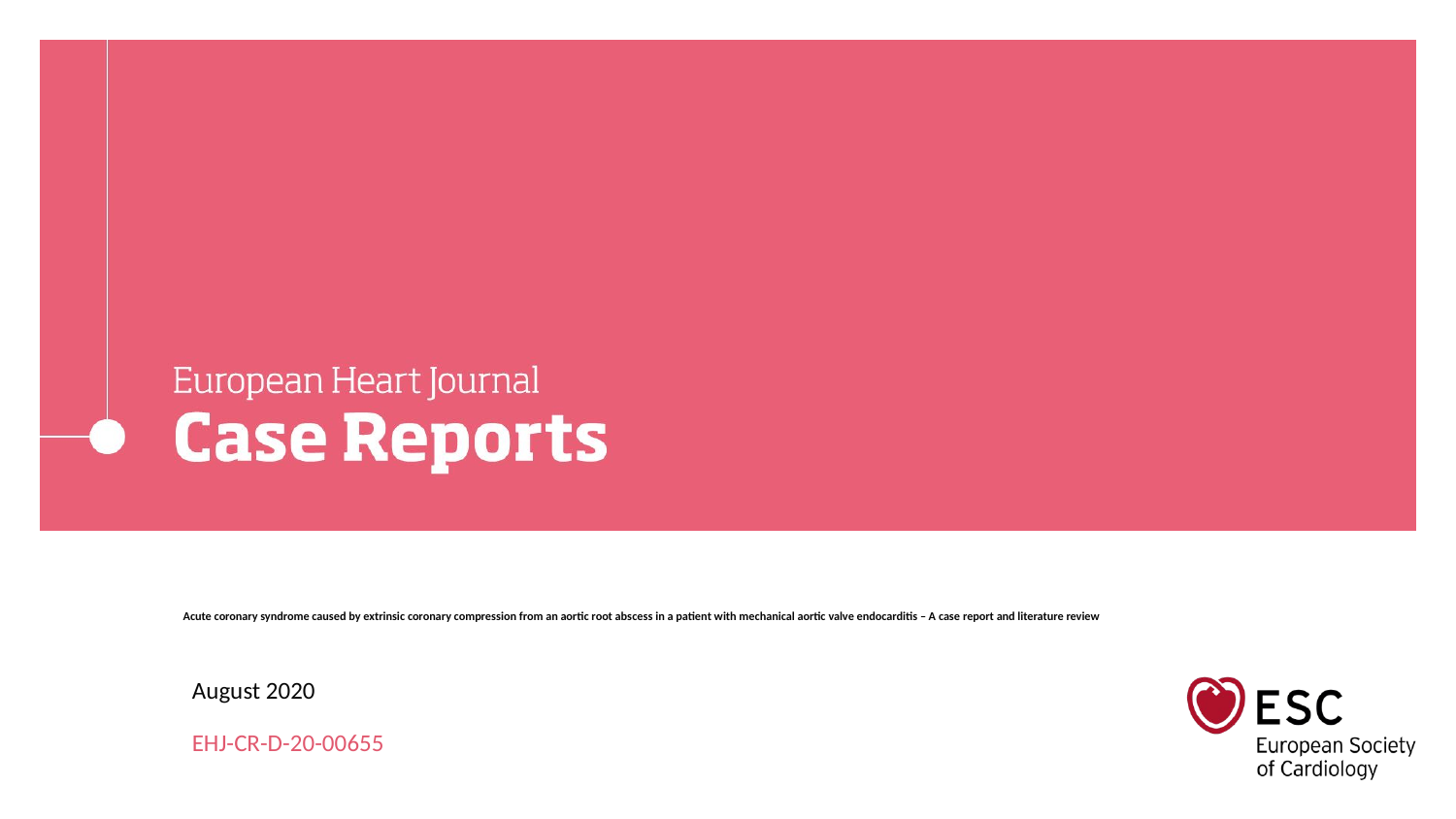

# Acute coronary syndrome caused by extrinsic coronary compression from an aortic root abscess in a patient with mechanical aortic valve endocarditis – A case report and literature review
August 2020
EHJ-CR-D-20-00655

## Slide 2
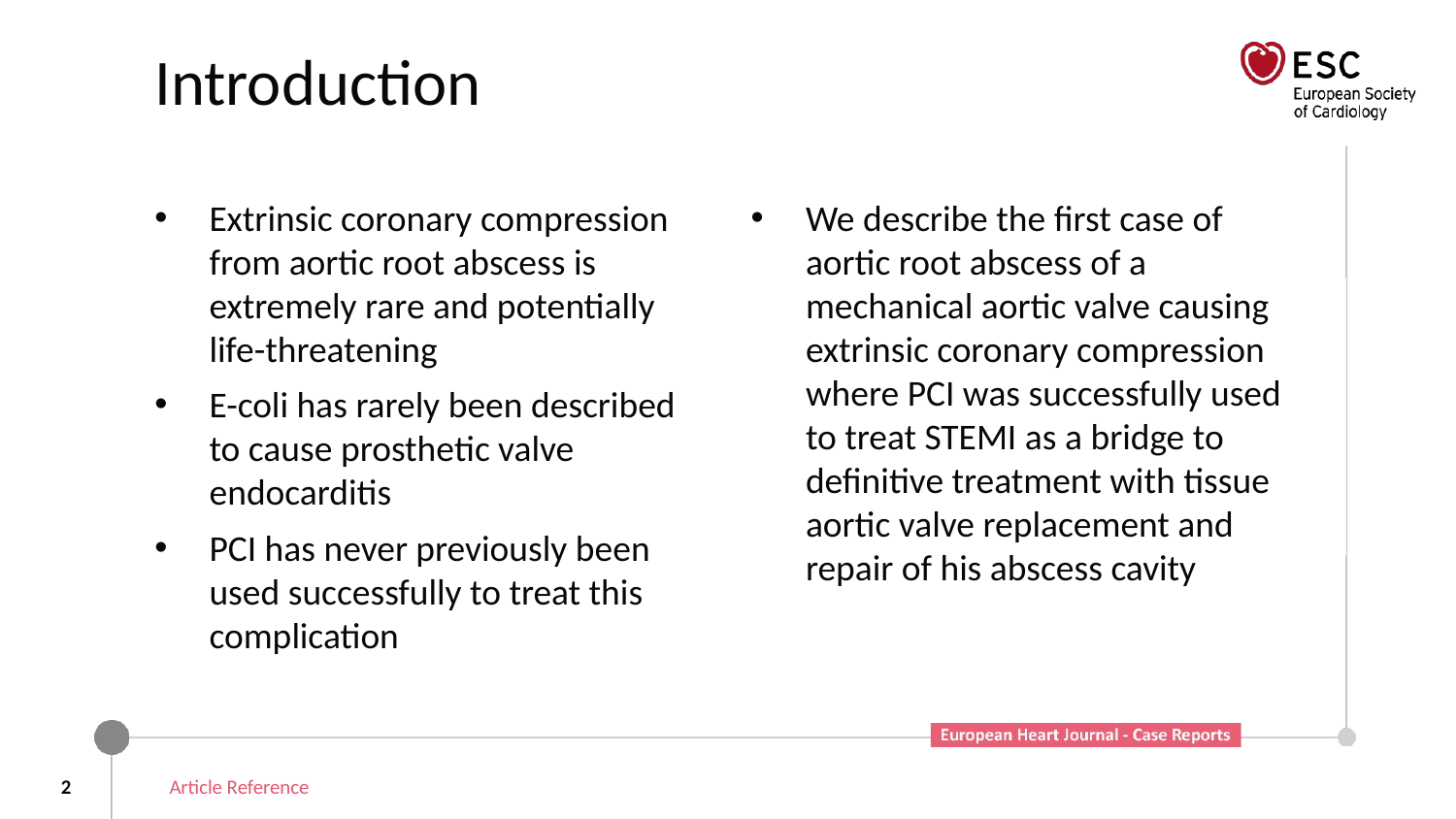

# Introduction
Extrinsic coronary compression from aortic root abscess is extremely rare and potentially life-threatening
E-coli has rarely been described to cause prosthetic valve endocarditis
PCI has never previously been used successfully to treat this complication
We describe the first case of aortic root abscess of a mechanical aortic valve causing extrinsic coronary compression where PCI was successfully used to treat STEMI as a bridge to definitive treatment with tissue aortic valve replacement and repair of his abscess cavity
2
Article Reference

## Slide 3
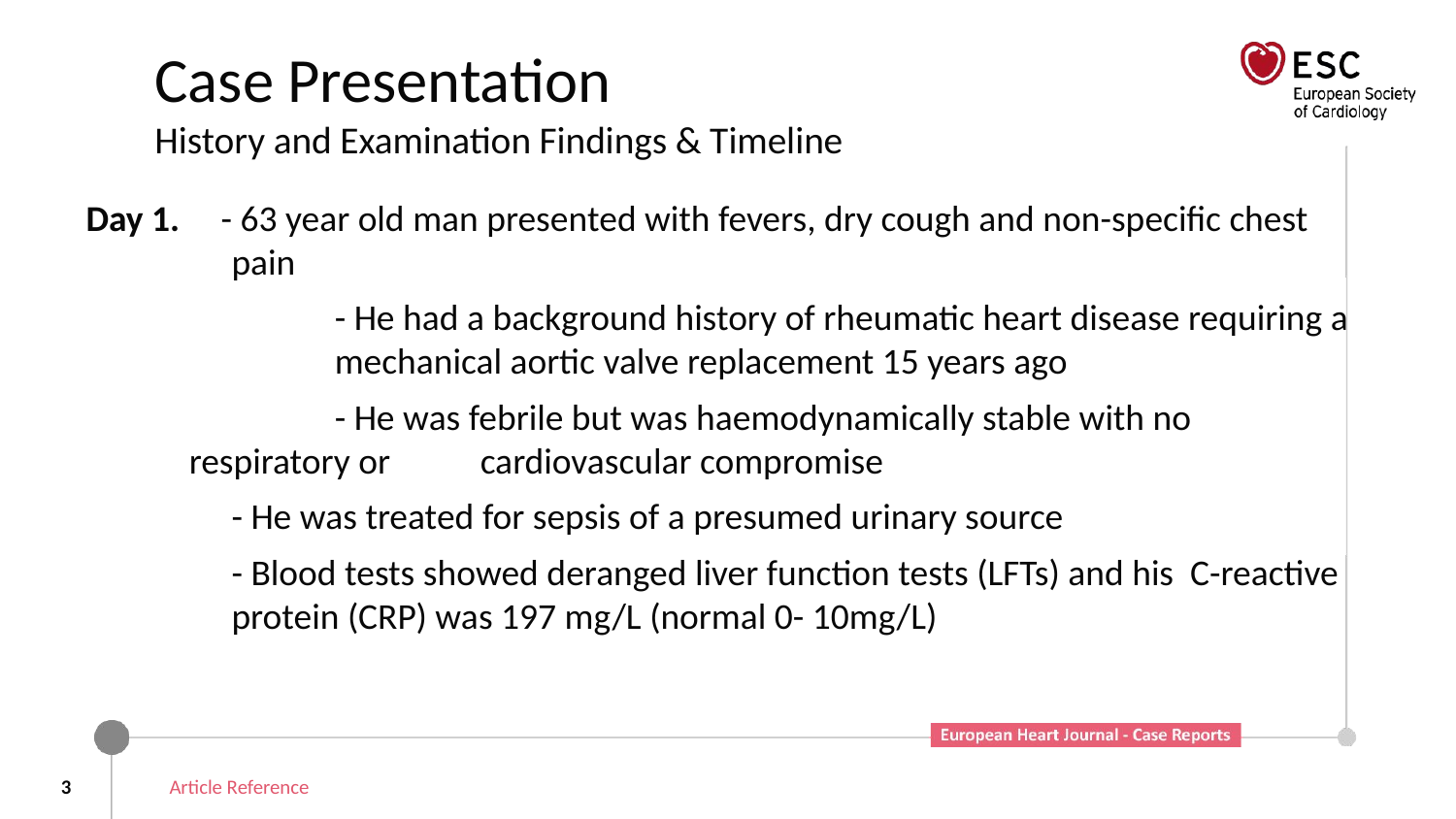

# Case Presentation History and Examination Findings & Timeline
Day 1. - 63 year old man presented with fevers, dry cough and non-specific chest 	pain
	- He had a background history of rheumatic heart disease requiring a 	mechanical aortic valve replacement 15 years ago
	- He was febrile but was haemodynamically stable with no 	respiratory or 	cardiovascular compromise
	- He was treated for sepsis of a presumed urinary source
	- Blood tests showed deranged liver function tests (LFTs) and his C-reactive 	protein (CRP) was 197 mg/L (normal 0- 10mg/L)
3
Article Reference

## Slide 4
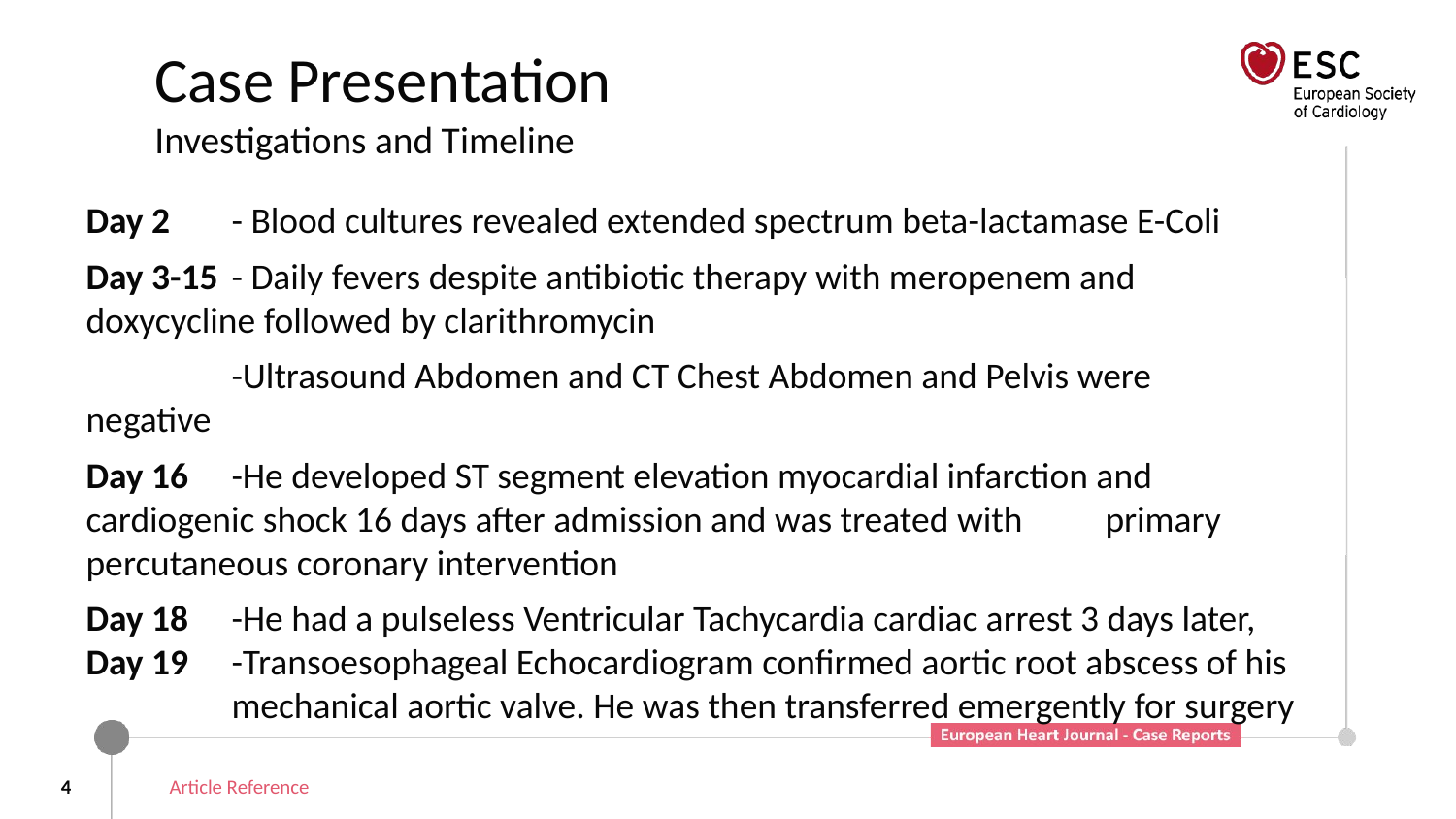

# Case Presentation Investigations and Timeline
Day 2	- Blood cultures revealed extended spectrum beta-lactamase E-Coli
Day 3-15	- Daily fevers despite antibiotic therapy with meropenem and 	doxycycline followed by clarithromycin
	-Ultrasound Abdomen and CT Chest Abdomen and Pelvis were 	negative
Day 16	-He developed ST segment elevation myocardial infarction and 	cardiogenic shock 16 days after admission and was treated with 	primary percutaneous coronary intervention
Day 18	-He had a pulseless Ventricular Tachycardia cardiac arrest 3 days later, Day 19	-Transoesophageal Echocardiogram confirmed aortic root abscess of his 	mechanical aortic valve. He was then transferred emergently for surgery
4
Article Reference

## Slide 5
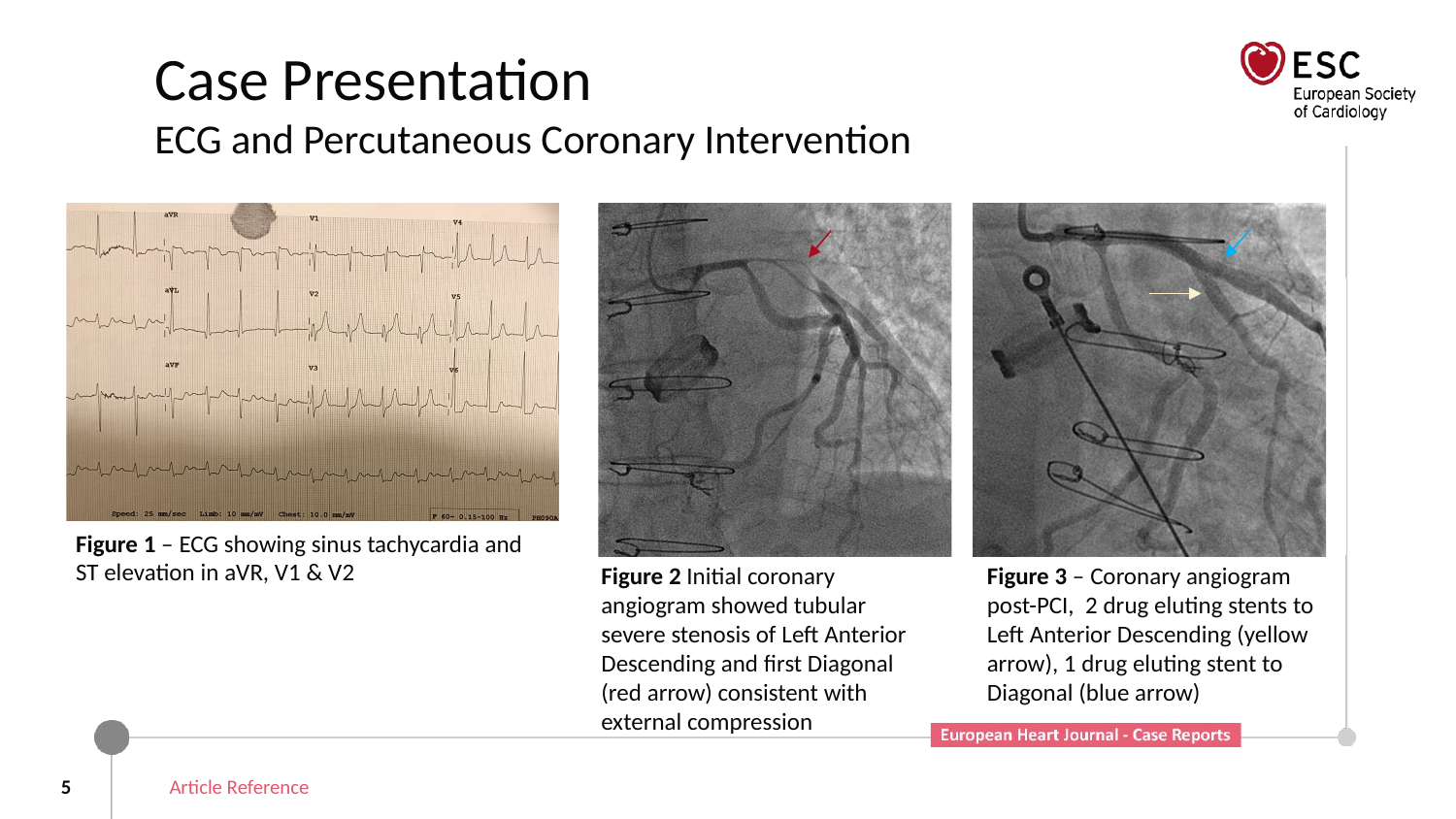

# Case Presentation ECG and Percutaneous Coronary Intervention
Figure 1 – ECG showing sinus tachycardia and ST elevation in aVR, V1 & V2
Figure 2 Initial coronary angiogram showed tubular severe stenosis of Left Anterior Descending and first Diagonal (red arrow) consistent with external compression
Figure 3 – Coronary angiogram post-PCI, 2 drug eluting stents to Left Anterior Descending (yellow arrow), 1 drug eluting stent to Diagonal (blue arrow)
5
Article Reference

## Slide 6
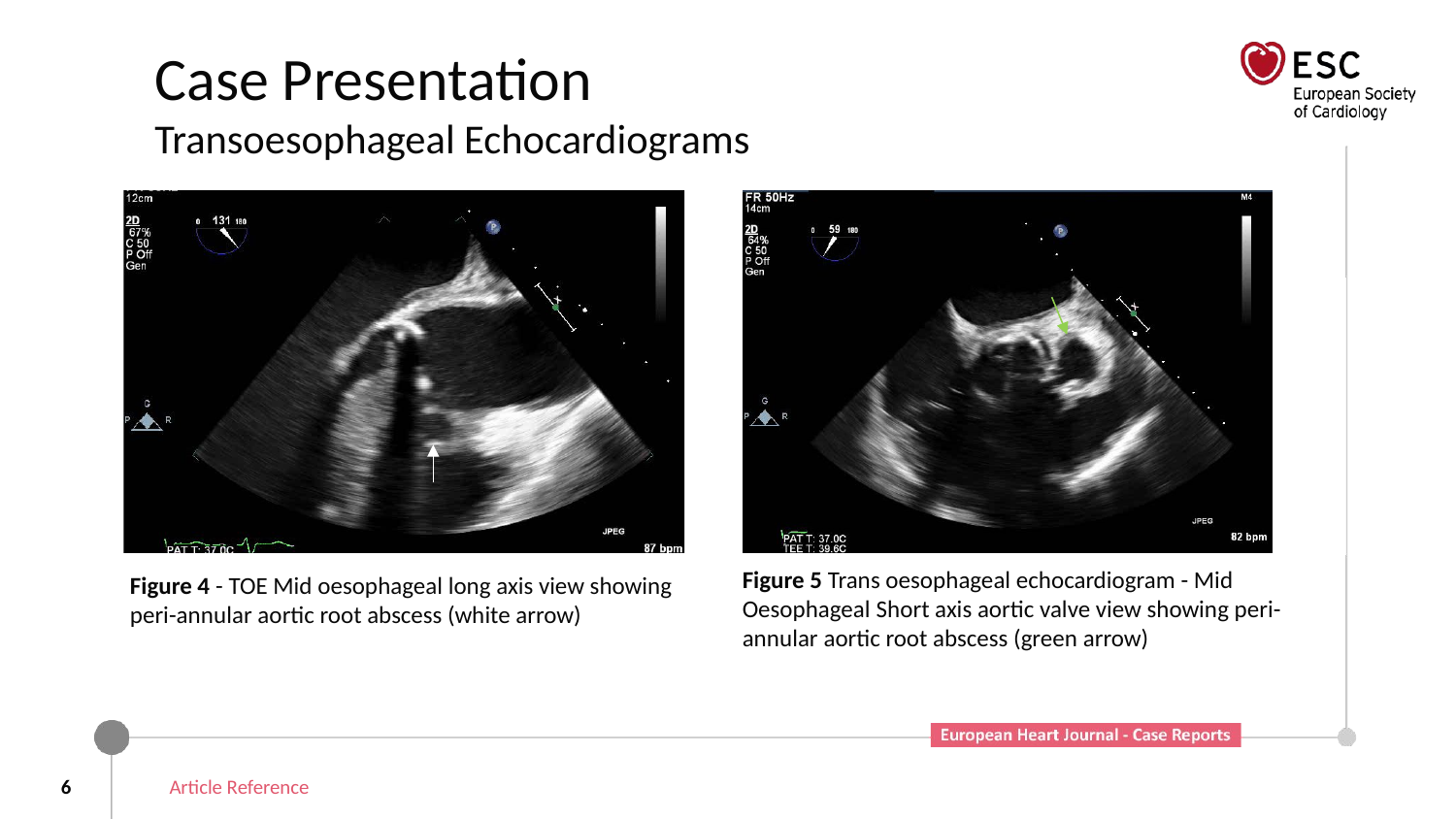

# Case Presentation Transoesophageal Echocardiograms
Figure 5 Trans oesophageal echocardiogram - Mid Oesophageal Short axis aortic valve view showing peri-annular aortic root abscess (green arrow)
Figure 4 - TOE Mid oesophageal long axis view showing peri-annular aortic root abscess (white arrow)
6
Article Reference

## Slide 7
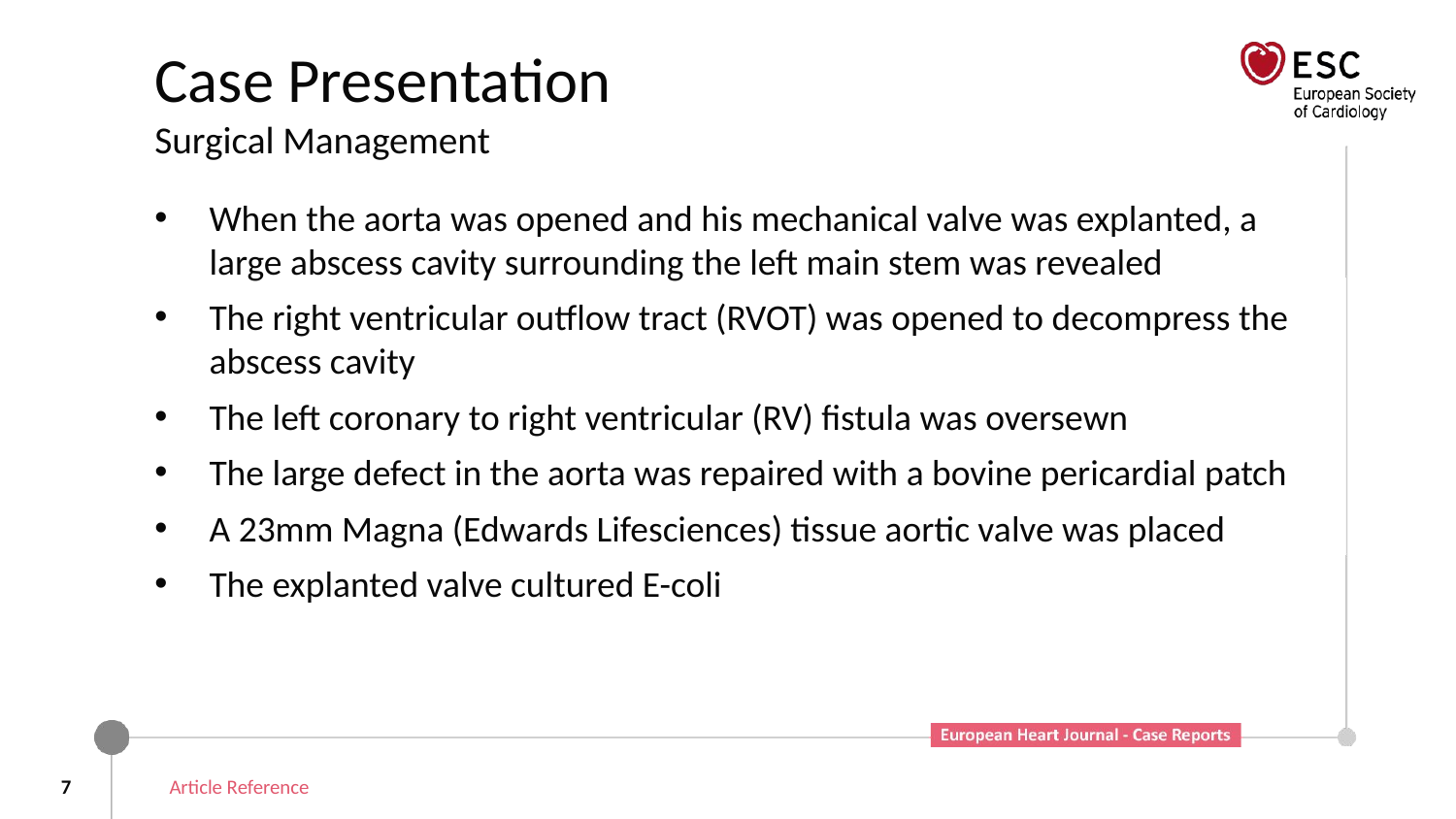

# Case Presentation Surgical Management
When the aorta was opened and his mechanical valve was explanted, a large abscess cavity surrounding the left main stem was revealed
The right ventricular outflow tract (RVOT) was opened to decompress the abscess cavity
The left coronary to right ventricular (RV) fistula was oversewn
The large defect in the aorta was repaired with a bovine pericardial patch
A 23mm Magna (Edwards Lifesciences) tissue aortic valve was placed
The explanted valve cultured E-coli
7
Article Reference

## Slide 8
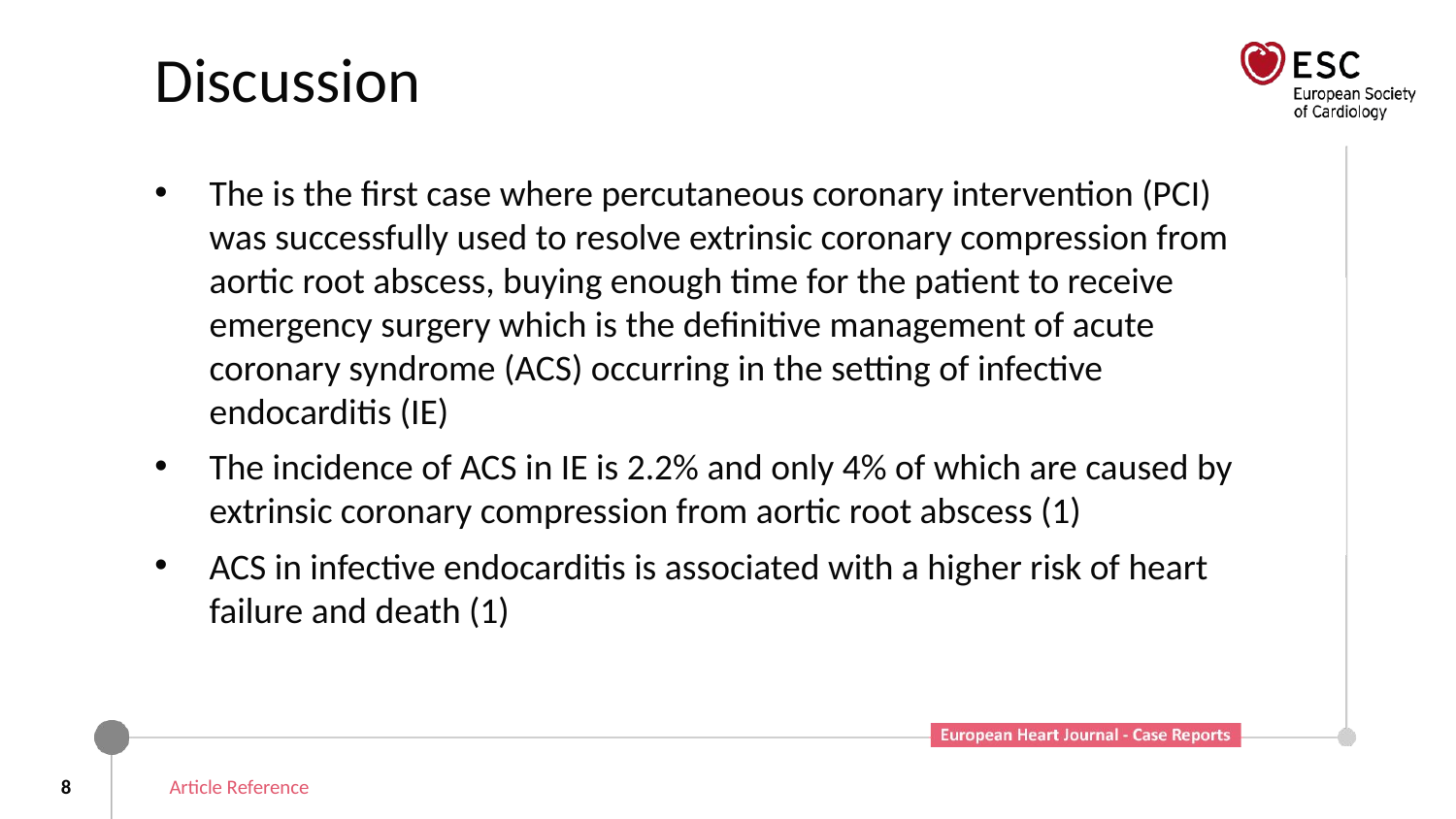

# Discussion
The is the first case where percutaneous coronary intervention (PCI) was successfully used to resolve extrinsic coronary compression from aortic root abscess, buying enough time for the patient to receive emergency surgery which is the definitive management of acute coronary syndrome (ACS) occurring in the setting of infective endocarditis (IE)
The incidence of ACS in IE is 2.2% and only 4% of which are caused by extrinsic coronary compression from aortic root abscess (1)
ACS in infective endocarditis is associated with a higher risk of heart failure and death (1)
8
Article Reference

## Slide 9
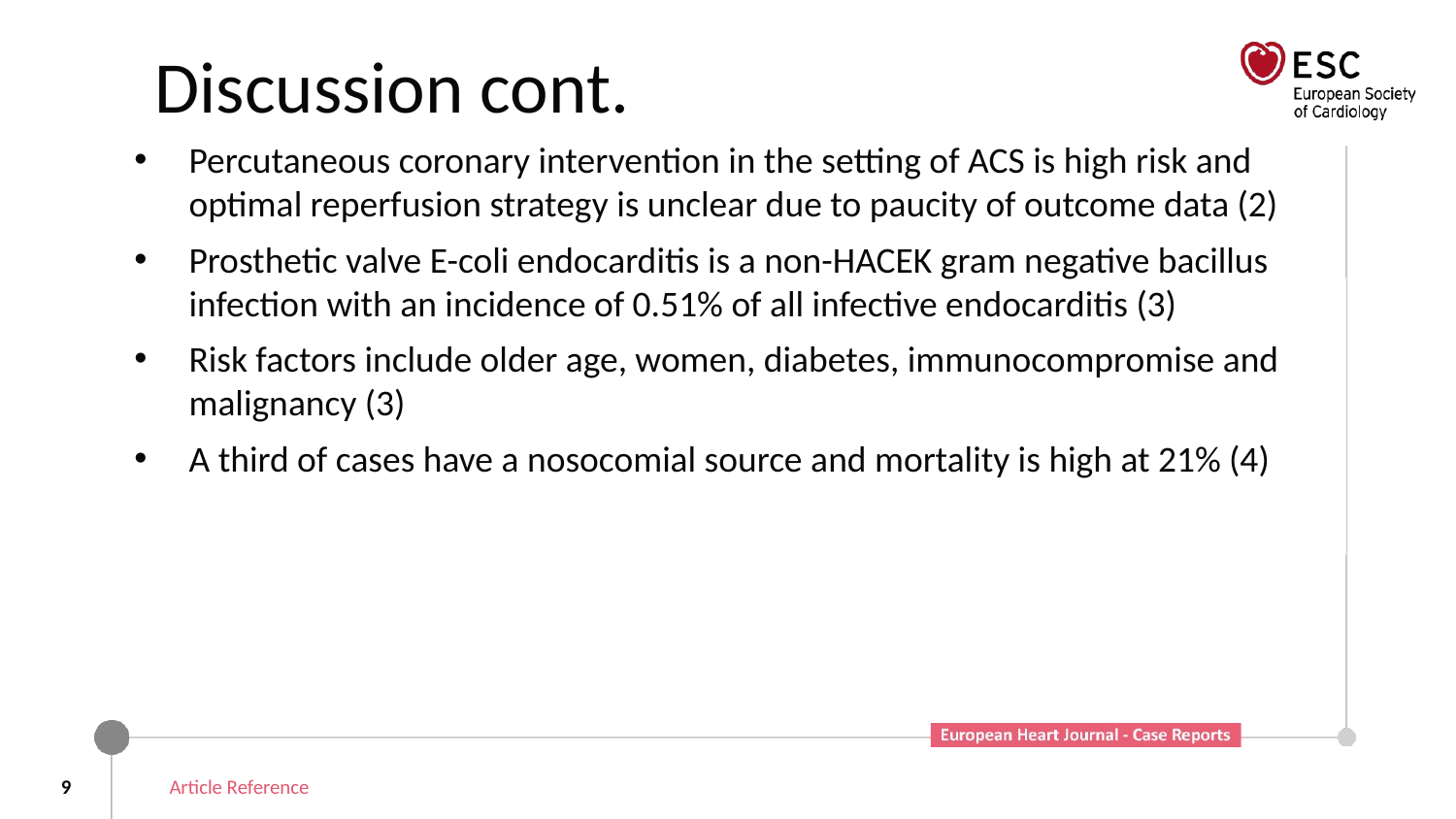

# Discussion cont.
Percutaneous coronary intervention in the setting of ACS is high risk and optimal reperfusion strategy is unclear due to paucity of outcome data (2)
Prosthetic valve E-coli endocarditis is a non-HACEK gram negative bacillus infection with an incidence of 0.51% of all infective endocarditis (3)
Risk factors include older age, women, diabetes, immunocompromise and malignancy (3)
A third of cases have a nosocomial source and mortality is high at 21% (4)
9
Article Reference

## Slide 10
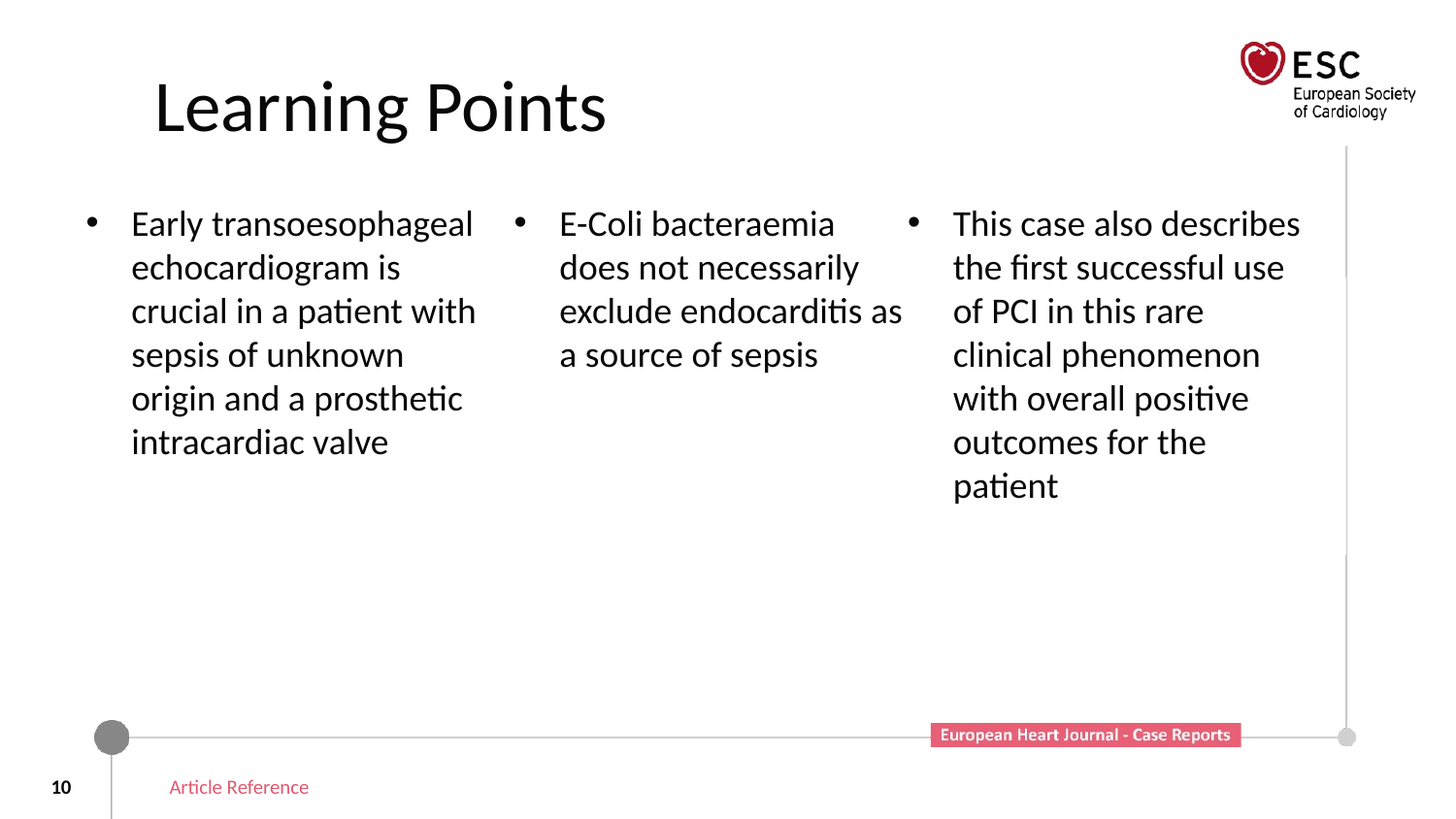

Learning Points
This case also describes the first successful use of PCI in this rare clinical phenomenon with overall positive outcomes for the patient
Early transoesophageal echocardiogram is crucial in a patient with sepsis of unknown origin and a prosthetic intracardiac valve
E-Coli bacteraemia does not necessarily exclude endocarditis as a source of sepsis
10
Article Reference

## Slide 11
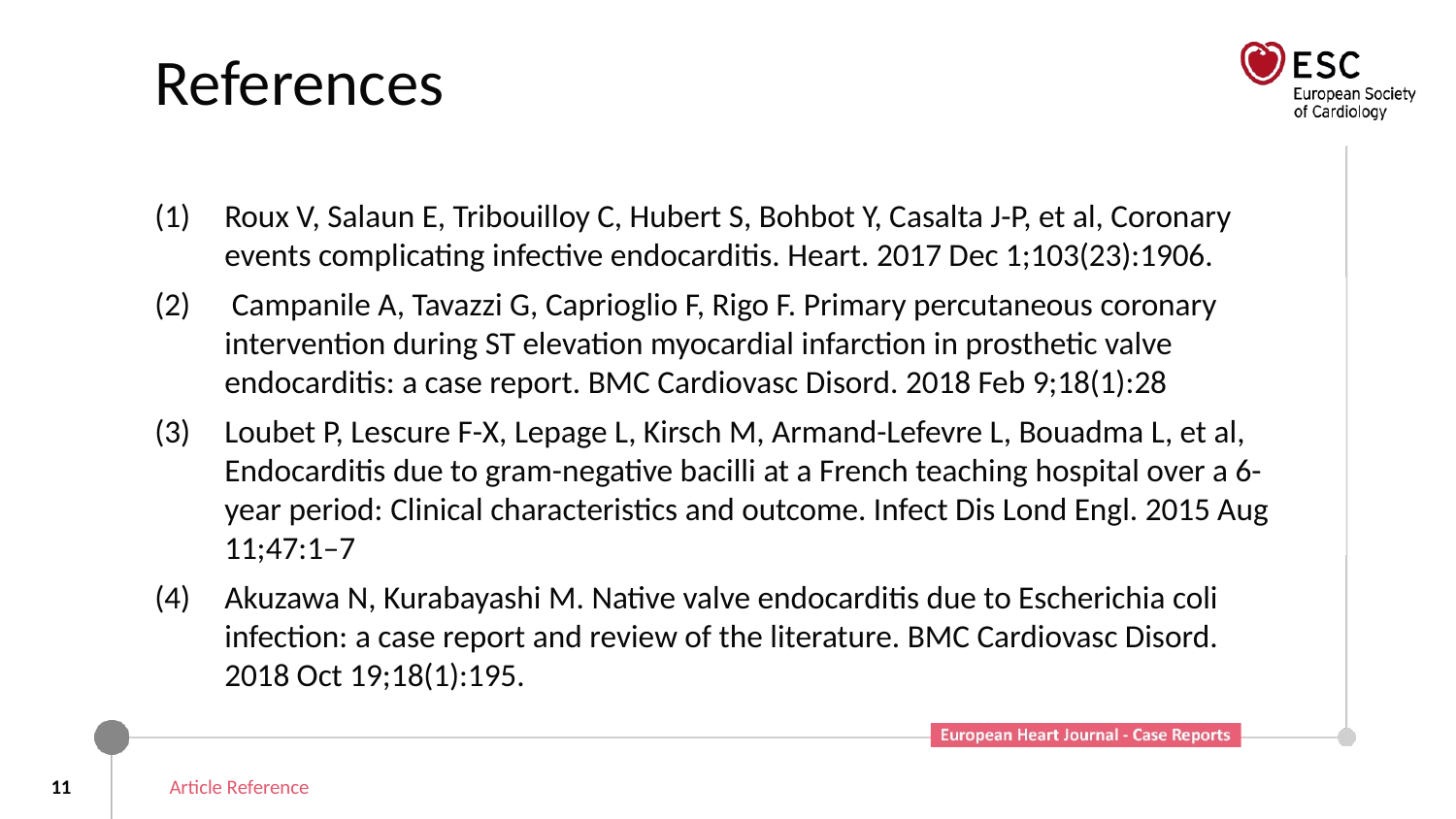

# References
Roux V, Salaun E, Tribouilloy C, Hubert S, Bohbot Y, Casalta J-P, et al, Coronary events complicating infective endocarditis. Heart. 2017 Dec 1;103(23):1906.
 Campanile A, Tavazzi G, Caprioglio F, Rigo F. Primary percutaneous coronary intervention during ST elevation myocardial infarction in prosthetic valve endocarditis: a case report. BMC Cardiovasc Disord. 2018 Feb 9;18(1):28
Loubet P, Lescure F-X, Lepage L, Kirsch M, Armand-Lefevre L, Bouadma L, et al, Endocarditis due to gram-negative bacilli at a French teaching hospital over a 6-year period: Clinical characteristics and outcome. Infect Dis Lond Engl. 2015 Aug 11;47:1–7
Akuzawa N, Kurabayashi M. Native valve endocarditis due to Escherichia coli infection: a case report and review of the literature. BMC Cardiovasc Disord. 2018 Oct 19;18(1):195.
11
Article Reference
